# TRITON and Beyond: New Insights into the Profile of Prasugrel

**DOI:** 10.1111/j.1755-5922.2011.00263.x

**Published:** 2011-02-17

**Authors:** Joseph A Jakubowski, Jeffrey S Riesmeyer, Sandra L Close, Amy G Leishman, David Erlinge

**Affiliations:** 1 Lilly Research Laboratories, Eli Lilly and CompanyIndianapolis, Indiana, USA; 2 Department of Cardiology, Lund University, Skane University HospitalLund, Sweden

**Keywords:** Acute coronary syndrome, Clopidogrel, Prasugrel, P2Y_12_, Thienopyridines

## Abstract

Prasugrel, a third-generation thienopyridine antiplatelet agent, demonstrated superior efficacy to clopidogrel but with an increased risk of bleeding in the phase III pivotal registration Trial to Assess Improvement in Therapeutic Outcomes by Optimizing Platelet Inhibition with Prasugrel–Thrombolysis in Myocardial Infarction (TRITON-TIMI 38). This article reviews and discusses select components of a large literature of prasugrel data that has emerged since the TRITON-TIMI 38 (TRITON) study primary disclosure.

## Introduction

Prasugrel is a novel member of the thienopyridine class of oral antiplatelet agents. It is rapidly converted *in vivo* to an active metabolite (AM) that binds specifically and irreversibly to the platelet P2Y_12_ purinergic receptor, thus inhibiting adenosine diphosphate (ADP)-mediated platelet activation and aggregation [1]. Prasugrel leads to platelet inhibition more rapidly and with less interindividual response variability compared to clopidogrel, resulting in an overall higher level of inhibition [2–5]. It is indicated to reduce the rate of thrombotic cardiovascular (CV) events (including stent thrombosis) in patients with acute coronary syndrome (ACS) who are to be managed with percutaneous coronary intervention (PCI).

Preclinical and clinical studies and the mechanistic basis for prasugrel's distinct antiplatelet profile have been the subject of a previous review article in this journal [5]. This current report reviews select components of a large body of literature on prasugrel that has emerged subsequent to the initial registration trial TRITON-TIMI 38 (TRITON) primary study disclosure [6]. The source of data for this review includes additional analyses from TRITON, i.e., prespecified subgroups, special populations, and additional pharmacodynamic (PD) and pharmacokinetic (PK) data from studies other than TRITON.

## Recent Data from TRITON Trial

### Early and Late Benefits of Prasugrel

**Figure 1:**
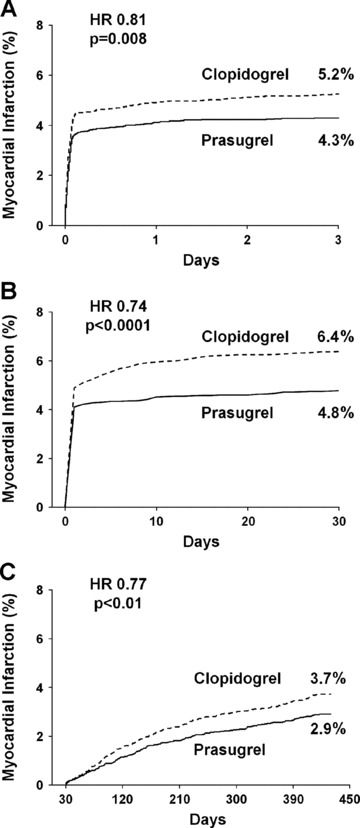
Landmark analyses of the Kaplan–Meier estimates of myocardial infarction in TRITON-TIMI 38. Myocardial infarction during the first 3 days after randomization (panel **A**), during the first 30 days after randomization (panel **B**), and from 30 days to the end of the study (panel **C**) (adapted with permission from [7, 8]).

Landmark analysis, a method of survival analysis that uses a fixed time after the start of treatment to determine the response to treatment, was used to assess the individual impact of the loading dose (LD) (60 mg prasugrel, 300 mg clopidogrel) and maintenance dose (MD) (10 mg prasugrel, 75 mg clopidogrel) phases on efficacy, safety, and net clinical benefit in TRITON. In TRITON, a significant reduction in the primary composite endpoint (death from CV causes, nonfatal myocardial infarction [MI], or nonfatal stroke) was seen in the prasugrel group both during the first 3 days (*P*= 0.01) and from day 3 to the end of the study (*P*= 0.003) compared to clopidogrel [6]. Further analyses of the component endpoint of MI [7,8] demonstrated that prasugrel, both LD and MD, significantly reduced the ischemic event of MI compared with the clopidogrel LD and MD during the very early phase (days 0–3), the early phase (days 0–30), and the later MD phase (days 30–450) of the study (Figure 1). As illustrated in Figure 1, the Kaplan–Meier curves separated during the first day, maintaining efficacy, and continued to separate throughout the 450-day follow-up period. Of note, the component endpoints of urgent target vessel revascularization (uTVR) and stent thrombosis (discussed later) were also significantly reduced both during the very early period and the maintenance phase with prasugrel treatment [7]. There was no significant increased bleeding in the very early period (*P*= 0.35), but a significant increase in bleeding in the later maintenance period (*P*= 0.036), although significant net clinical benefit (balance between the adverse effect of non-CABG TIMI major bleeding with the benefit of efficacy) with prasugrel was retained throughout [7]. These results are consistent with an ongoing benefit of prolonged treatment with prasugrel compared to clopidogrel and support the need for consistent platelet inhibition not only for the prevention of periprocedural ischemic events around the time of PCI, but also during long-term follow-up.

### Recurrent Cardiovascular Events

**Figure 2:**
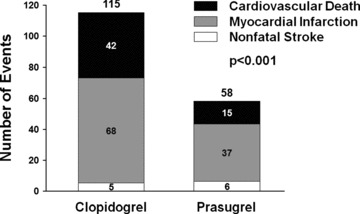
Additional primary endpoint events subsequent to initial event (modified from [9]).

In clinical trials patients who experience a primary endpoint are typically censored from the data analysis following the initial event with subsequent events not captured in the primary efficacy endpoint analysis. However, in a real-world setting, additional events occur and are clinically meaningful. Landmark analyses of TRITON were carried out to evaluate the risk of subsequent endpoint events following an initial nonfatal endpoint event for prasugrel versus clopidogrel [9]. Among patients with an initial nonfatal event, secondary events were significantly higher on clopidogrel compared to prasugrel (15.4 vs. 10.8%, hazard ratio [HR] 0.65, *P*= 0.016), thus resulting in an additional 115 events in the clopidogrel arm compared to 58 events in the prasugrel arm (*P* < 0.001) (Figure 2). Importantly, CV death following a nonfatal event was also significantly higher in the clopidogrel group (7.1 vs. 3.7%, HR 0.46, *P*= 0.008). Prasugrel was seen therefore to reduce not only the initial primary endpoint event compared with clopidogrel, but also subsequent events.

### Stent Thrombosis

**Figure 3:**
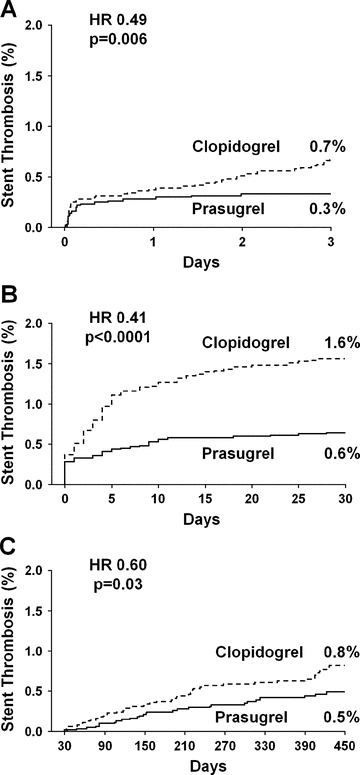
Landmark analyses of the Kaplan–Meier estimates of stent thrombosis. Stent thrombosis during the first 3 days after randomization (panel **A**), during the first 30 days after randomization (panel **B**), and from 30 days to the end of the study (panel **C**) (adapted with permission from [7,14]).

Coronary stenting, particularly with drug-eluting stents (DES), reduces restenosis in patients with ACS undergoing PCI [10]. However, their use carries a risk for developing stent thrombosis, a dangerous thrombotic complication with attendant mortality rates as high as 45%[11]. Stent thrombosis has been shown to be reduced by dual antiplatelet therapy [12,13]. In TRITON, 94% of patients (n = 12,844) received at least one stent (6461 bare-metal, 5743 DES). Of those who experienced stent thrombosis, death or MI was the outcome in 89% of patients, highlighting the severe consequences of stent thrombosis. In a prespecified endpoint analysis of stented patients, prasugrel was significantly more effective than clopidogrel in reducing ischemic events (CV death, nonfatal MI, or nonfatal stroke; 9.7 vs. 11.9%, HR 0.81, *P*= 0.0001) and stent thrombosis (1.13 vs. 2.35%, HR 0.48, *P* < 0.001) [14]. This effect was consistent across stent types: in DES (9.0 vs. 11.1%, HR 0.82, *P*= 0.019) and bare-metal stents (10.0 vs. 12.2%, HR 0.80, *P*= 0.003). As illustrated in Figure 3, it was evident that the reduction of stent thrombosis with prasugrel versus clopidogrel was greatest early (days 0–30), although late-stent thrombosis (days 30–450) was also statistically significantly reduced (*P*= 0.03). This greater reduction in stent thrombosis was also observed in the very early stage (days 0–3) [7].

### Prespecified Subgroups

A number of patient subgroup analyses of TRITON have been conducted. These were specified prior to database lock and unblinding of the data [6].

#### ST-Elevation Myocardial Infarction

Primary or secondary PCI is increasingly used in the treatment of ST-elevation myocardial infarction (STEMI) [15–17]. Accordingly, the efficacy and safety of prasugrel compared to clopidogrel in reducing ischemic events in this patient subgroup of TRITON were evaluated [18]. In the entire STEMI group (n = 3534) at 30 days, 9.5% receiving clopidogrel had met the primary composite endpoint of CV death, nonfatal MI, or nonfatal stroke, compared with 6.5% of patients receiving prasugrel (HR 0.68, *P*= 0.0017). This effect was seen to continue to 15 months (12.4 vs. 10.0%, HR 0.79, *P*= 0.0221). The key secondary endpoint (composite of CV death, nonfatal MI, or nonfatal uTVR) was significantly higher at 30 days (8.8 vs. 6.7%, HR 0.75, *P*= 0.0205) and at 15 months (12.0 vs. 9.6%, HR 0.79, *P*= 0.0250) with clopidogrel compared with prasugrel, as was CV death at 30 days (2.4 vs. 1.4%, HR 0.61, *P*= 0.0469). No differences in bleeding were seen between treatment groups, at 30 days (*P*= 0.3359) or 15 months (*P*= 0.6451). However, TIMI major bleeding in the small number of patients (4%) who underwent coronary artery bypass graft (CABG) surgery was significantly increased with prasugrel (18.8 vs. 2.7%, odds ratio 8.19, *P*= 0.0033). Even when CABG-related bleeding events were considered, the net clinical benefit significantly favored prasugrel compared with clopidogrel at both 30 days and 15 months. The beneficial effects of prasugrel were seen without an excess in bleeding in the primary and even more prominently in the secondary PCI group [18].

#### Diabetes

Ironically, ACS patients with diabetes mellitus (DM) are characterized by increased platelet reactivity and yet have a reduced antiplatelet response to clopidogrel [19–23]. Therefore, it is of interest to consider the relative efficacy of prasugrel versus clopidogrel in this population at high risk of CV events [24]. Prasugrel and clopidogrel were compared in the 3146 DM patients enrolled in TRITON [25] and showed that DM patients treated with prasugrel had a significant reduction in the primary composite endpoint of CV death, nonfatal MI, or nonfatal stroke compared to clopidogrel (12.2 vs. 17.0%, HR 0.70, *P* < 0.001), largely driven by a lower incidence of MI (40% relative risk reduction). Interestingly, non-CABG TIMI major bleeding events were similar for prasugrel and clopidogrel (2.5 vs. 2.6%, HR 1.06, *P*= 0.81) with a resulting greater net clinical benefit than in the overall TRITON population. These findings suggest that prasugrel may in particular benefit DM patients.

**Figure 4:**
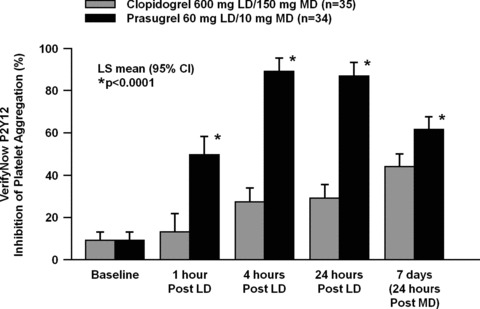
Platelet function assessed by VerifyNow P2Y12 in coronary artery disease patients with type 2 diabetes mellitus (modified from [30]).

It has been suggested that higher doses of clopidogrel (600 mg LD/150 mg MD) may overcome the decreased response to this agent in DM patients [26–28]. Erlinge et al. 2008 [29] found that prasugrel (60 mg/10 mg) in DM patients with stable CAD provided more effective platelet inhibition than clopidogrel (600 mg/150 mg). In a separate PD study, OPTIMUS-3, which compared the effects of prasugrel (60 mg/10 mg) with double dose clopidogrel (600 mg/150 mg) in patients with type 2 DM and CAD [30], the level of platelet inhibition, as assessed by the point-of-care test VerifyNow P2Y12, was significantly greater for prasugrel as early as 1 h after dosing and at all time-subsequent points (*P* < 0.0001) (Figure 4). These results confirm the hypothesis that treatment of DM patients with prasugrel is associated with greater platelet inhibition than that found with double dose clopidogrel.

#### Pharmacogenomics

Both clopidogrel and prasugrel require biotransformation to AMs each with varying degrees of dependence on members of the cytochrome P450 (CYP) family of oxidative enzymes. The genes that encode the CYP enzymes are polymorphic, with certain variations resulting in reduced enzymatic function and consequent effects on exposure [31]. The platelet-inhibitory response to clopidogrel demonstrates substantial interpatient variability [32–34], and patients with reduced levels of platelet inhibition have been shown to be at increased risk of CV events [35–39]. Using data collected from the TRITON genetic substudy, carriers of a reduced-function CYP2C19 allele treated with clopidogrel had significantly higher rate of CV events, including stent thrombosis, than did noncarriers [40]. This observation has been confirmed by several other groups [41,42] and by meta-analyses [43–45]. Consistent with these clinical observations, CYP2C19 reduced-function variants have also been associated with lower levels of clopidogrel AM and reduced platelet inhibitory response [38,40,46]. In contrast, for patients randomized to prasugrel in the TRITON genetic substudy (n = 1466), none of the common genetic variations in the CYPs involved in the metabolism of either drug (including CYP2C19) influenced the low rate of CV events associated with prasugrel [47]. Consistent with this lack of effect on prasugrel's efficacy, other studies found no significant association between individual reduced-function CYP genes and AM exposure or platelet response for prasugrel [46,47]. In summary, the observed differences in pharmacological responses between clopidogrel and prasugrel can in part be explained by genetic variation, differential metabolic pathways, and differing exposure to AMs.

In addition to the CYPs, genes involved in the transport of drugs have increasingly been recognized to play an important role in a drug's pharmacological clinical profile [48]. P-glycoprotein, an efflux protein, has been shown to influence clopidogrel absorption [49]. A recent clinical study has suggested that patients with a T allele in the 3435 variant of ABCB_1_, a gene which codes P-glycoprotein, have an increased rate of CV events [42]. In the 2932 patients from the TRITON genetic subgroup, those treated with clopidogrel and homozygous for the ABCB_1_ 3435T allele had a 72% increased risk of the composite primary endpoint of CV death, nonfatal MI, or nonfatal stroke (*P*= 0.002) compared with patients who did not carry this polymorphism [50]. The prasugrel group showed a trend toward an increased risk of primary endpoint; however, this was not statistically significant [50]. Taken together these data highlight that patient subgroups, as defined by certain genetic variation, may gain particular benefit from treatment with prasugrel.

## Additional PD and PK Studies with Clinical Implications

### Drug–Drug Interactions (DDIs)

No clinically significant DDIs have been identified with prasugrel including aspirin (75–325 mg/day), heparin, GPIIb/IIIa inhibitors, statins, digoxin, and drugs that elevate gastric pH, including proton pump inhibitors and H2 blockers [51]. In contrast, there is evidence that proton-pump inhibitors (PPIs), in particular omeprazole, diminish the antiplatelet effect of clopidogrel and worsen clinical outcome [52]. However, there is conflicting evidence on whether the impact of PPIs on PK/PD adversely affect clinical outcomes [53,54]. In post hoc analysis using data from two randomized studies (PRINCIPLE-TIMI 44 and TRITON) which compared prasugrel with clopidogrel, no association between PPI use and risk of the primary endpoint (CV death, MI, or stroke) for patients treated with either agent was found [55]. Other studies assessing DDIs with prasugrel and clopidogrel found that ketoconazole, a CYP3A inhibitor, while reducing generation of clopidogrel's AM and its platelet-inhibitory effects, did not have a significant effect on prasugrel [56]. In an open-label randomized study of prasugrel plus aspirin, prasugrel coadministered with aspirin led to a greater platelet inhibition than aspirin alone [57]. Further information on DDIs with prasugrel is available in a recent review article [58].

### “Rebound” Following Thienopyridines

Various studies have demonstrated a clustering of thrombotic events after discontinuation of treatment with thienopyridines [59,60]. It has been speculated that these could in part be due to a rebound effect on platelets after withdrawing clopidogrel treatment, leading to a hyperthrombotic period where platelets have heightened reactivity compared to that before thienopyridine treatment [60–62]. In a double-blind randomized study to assess platelet rebound after abrupt cessation of clopidogrel treatment and its attenuation by clopidogrel tapering, no difference was found between the two treatment groups [63]. Results from the recent PACT study [64] found no evidence of a rebound in platelet reactivity associated with clopidogrel cessation. The potential for a rebound in platelet reactivity following prasugrel was prospectively studied in the OPTIMUS-3 (see above) study. Rebound was prospectively defined as a ≥20% increase from baseline reactivity measured 6–8 days following cessation of study drug. No consistent evidence of a rebound effect with prasugrel (or clopidogrel) was found [30,65]. This lack of rebound in platelet reactivity following prasugrel was also noted in separate studies of healthy subjects and those with end-stage renal disease [66]. These results suggest that the increase in thrombotic events observed following thienopyridine withdrawal may simply be the result of treatment cessation and restoration of platelet function rather than the development of platelet hyperreactivity. It is probable that areas at risk for thrombosis, such as ulcerated plaques and exposed stents, are protected during thienopyridine treatment and thrombosis occurs when this is discontinued.

### Switching among Thienopyridines

**Figure 5:**
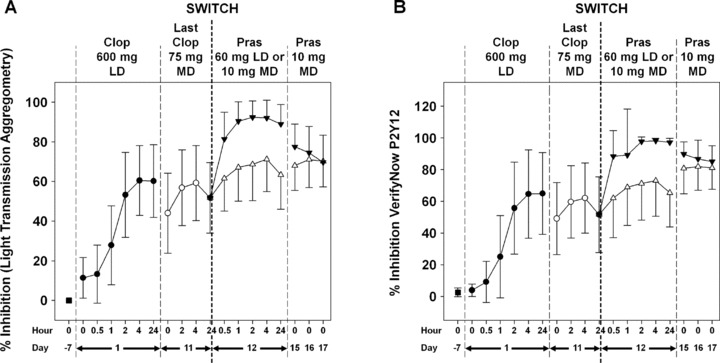
The impact of switching from clopidogrel to prasugrel as determined by traditional and point-of-care testing. Mean percent platelet inhibition of platelet aggregation (% inhibition) as measured by light transmission aggregometry in response to 20 μM ADP (panel **A**). Mean percent platelet inhibition (% inhibition) reported by VerifyNow P2Y12 (panel **B**). Clop = clopidogrel; Pras = prasugrel. (Modified from[68].)

As new antiplatelet agents are approved, there will be an increasing need to evaluate the consequence of switching among therapies without a wash-out interval. A pilot study that examined switching directly from MD clopidogrel to MD prasugrel (10 mg MD, with or without a 60 mg LD) was carried out in aspirin-treated healthy subjects [67,68]. As illustrated in Figure 5, the presence of clopidogrel did not affect the ability of prasugrel to further inhibit platelet aggregation with the lower levels of inhibition reaching steady state within 4–5 days of switching, as demonstrated by light transmission aggregation and confirmed by VerifyNow P2Y12. The data also showed that pretreatment with clopidogrel did not impact the ability of a prasugrel LD to provide immediate high-grade inhibition. The transition was well tolerated and not associated with an increase in bleeding events, albeit in a healthy population. The SWAP study which evaluated the PD response in patients with a recent history of ACS (n = 128) on MD clopidogrel therapy (75 mg) who switched to prasugrel MD [69], found that prasugrel maintained its ability to further suppress platelet aggregation and that this could be achieved as early as 2 h after the administration of a prasugrel 60 mg LD, with no major safety events observed. Further studies in stable CAD patients undergoing planned PCI in PRINCIPLE-TIMI 44 [70] and in ACS patients in ACAPULCO [71], demonstrated a significantly greater antiplatelet effect with prasugrel after switching directly from high dose clopidogrel (600 mg/150 mg). Platelet-inhibition data from these switching studies consistently demonstrate that prior clopidogrel treatment does not appear to impede prasugrel's antiplatelet effect as has been observed when switching directly from a direct-acting P2Y_12_ antagonist [72], and that switching directly to prasugrel has been well tolerated.

#### Safety

In TRITON, the key safety endpoint of non-CABG TIMI major bleeding was higher with prasugrel compared to clopidogrel (2.4 vs. 1.8% respectively, HR 1.32, *P*= 0.03) [6]. In this review, bleeding has been described where it substantially differed from that seen in the overall TRITON population. Two groups in which no statistically significant bleeding differences were observed between prasugrel and clopidogrel were STEMI and DM patients. One group in TRITON where major bleeding on prasugrel was notable was in patients undergoing CABG; however, post hoc analysis indicated that when prasugrel was withheld for 7 days before surgery, major bleeding was substantially reduced [51]. Patients with a previous stroke/transient ischemic attack (TIA) were found to have a higher risk of bleeding and were contraindicated from treatment with prasugrel [51].

In TRITON, both very elderly (≥75 years) and low body weight (<60 kg) patients experienced higher rates of bleeding, for both prasugrel and clopidogrel [6]. From the TRITON PK substudy (n = 1159) [73], statistical modeling revealed that body weight was the most significant patient characteristic influencing exposure to prasugrel's AM, with patients <60 kg experiencing 30% higher mean exposure than patients ≥60 kg. Age was also a factor, with mean prasugrel AM exposures for patients ≥75 years 19% higher compared with patients <75 years. Importantly, unlike bleeding, efficacy did not change across the AM exposure range; thus dose reduction should maintain efficacy and reduce bleeding [74]. In view of the above data, a MD adjustment from 10 to 5 mg is recommended for these patient populations. The validity of this approach is being tested in ongoing PK/PD studies of very elderly (GENERATIONS) and low body weight patients (FEATHER) (http://www.clinicaltrials.gov NCT01107912 and NCT01107925, respectively). A further ongoing study (TRILOGY ACS) [75], in medically managed patients, will also include an assessment of the clinical benefit of the prasugrel 5 mg MD in the low body weight and very elderly populations.

When patients without a history of stoke/TIA, who weigh <60 kg, or who are ≥75 years, are excluded from the overall TRITON population, TIMI major bleeding was not significantly different between prasugrel and clopidogrel (*P*= 0.17), but the greater efficacy of prasugrel (CV death/MI/nonfatal stroke) was maintained (11.0 vs. 8.3%, HR 0.74, *P* < 0.001) [76].

In PK/PD studies, it was also noted that the mean exposure to prasugrel AM and platelet inhibition were higher in Asian subjects compared to Caucasian subjects [77,78]. An integrated analysis of PK data suggested that these exposure differences were driven by a disproportionate impact of low body weight subjects in the Asian group, especially those <60 kg. Based on current recommendations where low body weight patients will receive a lower MD of prasugrel, these observed differences should not have clinical consequences [79].

Recent commentaries have raised questions regarding prasugrel and the risk of cancer [80–82]. As reported in the primary disclosure of the TRITON results [6], colonic neoplasm was diagnosed in 0.2% of prasugrel patients and 0.1% of clopidogrel patients (*P*= 0.03). Newly diagnosed malignancies were reported in 1.6% and 1.2% of patients treated with prasugrel and clopidogrel respectively [51]. It was unclear if these observations were causally related, random occurrences, or detection bias. Subsequent analysis of nonbenign neoplasms diagnosed after the start of study medication in TRITON, and according to randomization strategy, found no significant difference (*P*= 0.30) in the rate of new cancers with prasugrel versus clopidogrel [83]. The Food and Drug Administration (FDA) concluded that any difference between treatment arms was likely due to chance, and the prasugrel advisory committee was in agreement [84]. All FDA conclusions related to this topic may be found in the summary documents of the prasugrel action package [85] and prescribing information [51]. It should be noted that TRITON was not prospectively designed to answer questions related to cancer risk. Further data pertaining to cancer are currently being prospectively collected in the ongoing TRILOGY ACS study [75], which will allow further analysis of the relationship.

## Conclusion

Since the primary disclosure of prasugrel's pivotal phase III trial, TRITON-TIMI 38, knowledge concerning the efficacy, safety, net clinical benefit, and underlying mechanisms of prasugrel has increased substantially. From in-depth analyses of TRITON and other studies, it has become apparent that a number of subpopulations appear to have a better benefit/risk profile for prasugrel than the overall population, including STEMI patients and those with diabetes. The population, indicated to receive a 10 mg MD of prasugrel (patients without a history of stroke/TIA, age <75 years, and weight ≥60 kg), is observed to have a smaller bleeding increment over clopidogrel, but at no cost to efficacy, than the overall TRITON population. This has led to the recommendation of a lower maintenance dose (5 mg) for very elderly and low body weight patients. The pharmacogenomic substudy of TRITON has contributed to a shift in focus toward personalized medicine, with genetic and functional testing likely playing a key future role [86]. With the advent of new P2Y_12_ antagonists such as prasugrel and ticagrelor, and proteinase-activated receptor 1(PAR-1) antagonists under development (e.g., vorapaxar and atopaxar), the choice of agents and approaches to management of ACS will ultimately increase and will hopefully benefit patients.
